# Crisis responders' perspectives on animal-assisted crisis response in acute trauma settings: a qualitative study

**DOI:** 10.1080/17482631.2026.2732305

**Published:** 2026-09-10

**Authors:** Batya G. Jaffe

**Affiliations:** a Wurzweiler School of Social Work, Yeshiva University, New York, USA

**Keywords:** Animal-assisted crisis response, trauma, psychological first aid, qualitative research, crisis response

## Abstract

**Objective:**

Animal-Assisted Crisis Response (AACR) is used to provide immediate psychosocial support following traumatic events, yet limited qualitative research has examined how crisis responders understand its role in acute trauma settings. This study explored responders’ perspectives on the relational, emotional, and operational dimensions of AACR.

**Method:**

Semi-structured interviews and demographic questionnaires were completed with 21 crisis responders involved in acute trauma response. Data were analysed using reflexive thematic analysis supported by ATLAS.ti.

**Results:**

Four themes were identified. Participants described AACR as facilitating relational and non-verbal engagement, emotional regulation, grounding, and perceived safety. They also reported that AACR could influence the emotional and interpersonal dynamics of trauma scenes and support responders and bystanders. Implementation was shaped by cultural attitudes toward dogs, scene conditions, organisational requirements, handler preparedness, and animal welfare considerations.

**Conclusions:**

The findings provide qualitative insight into how crisis responders perceive AACR within acute trauma settings. AACR was described as a potentially valuable component of psychosocial crisis response, particularly when verbal engagement is difficult. Further research should examine survivor experiences, culturally responsive implementation, responder well-being, animal welfare, and the conditions under which AACR may be appropriate.

## 1. Introduction

Traumatic events can overwhelm individuals’ usual coping capacities, disrupting their sense of safety, control, connection, and meaning while inducing psychological distress, anxiety, vulnerability, and confusion (American Psychiatric Association, 2013; van der Kolk, 2014). Survivors frequently experience acute stress and grief in the immediate aftermath of trauma, highlighting the importance of early psychosocial responses that promote stabilization and support adaptive coping (Eaton-Stull & Flynn, 2015; Calhoun et al., 2022). These early interventions play an important role in supporting health and well-being during the acute stages of trauma response. Recent systematic reviews have noted that, despite the widespread use of early psychosocial interventions such as Psychological First Aid, robust evidence supporting their effectiveness remains limited, highlighting the need for further evaluation of early trauma response approaches (Hermosilla et al., 2023).

Recent evidence also suggests that important questions remain regarding the implementation and delivery of early psychosocial interventions in real-world trauma settings (Wang et al., 2024). During crises—events in which individuals’ usual coping mechanisms are overwhelmed (Caplan, 1964)—immediate psychosocial interventions aim to promote stabilization and support adaptive coping following traumatic events. Animal-Assisted Crisis Response (AACR) is a specialized form of Animal-Assisted Intervention (AAI) developed for crisis and disaster settings, in which trained responders and therapy dogs are incorporated into psychosocial crisis response (HOPE Animal-Assisted Crisis Response, 2000; Greenbaum, 2006). Rather than functioning as a standalone treatment, the therapy dog is incorporated into the responder’s interaction with survivors and others affected by a crisis. The animal’s presence has been described as helping responders establish rapport, provide a calming and non-threatening presence, facilitate communication, and create opportunities for emotional connection during crisis response (Greenbaum, 2006; Eaton-Stull, 2014; Eaton-Stull et al., 2024). Existing literature therefore suggests that AACR may facilitate comfort, reduce anxiety, promote perceived safety, and support trauma-informed crisis care; however, the empirical evidence base remains limited (Graham, 2009; Bua, 2013; Eaton-Stull, 2014).

### 1.1. AACR as early psychosocial intervention

A significant milestone in the development of AACR occurred with the publication of the United States AACR National Standards protocol in 2010 (Eaton-Stull et al., 2010), which established a standardized framework for its use in crisis settings (Stewart et al., 2016). AACR involves trained responders and therapy dogs within trauma and emergency settings with the aim of providing immediate psychosocial support to survivors, first responders, and affected communities. To date, AACR has been implemented across a range of settings, including disasters, mass-casualty incidents, and other traumatic events affecting communities (Eaton-Stull & Flynn, 2015). The literature has increasingly explored its potential role in supporting the emotional and relational dimensions of crisis response (Bua, 2013).

### 1.2. AACR and psychosocial support

Existing literature suggests that AACR may provide psychosocial support in trauma and mental health contexts (O’Haire et al., 2015). AACR has been described as helping to “establish rapport, build therapeutic bridges, normalize the experience, and act as a calming agent or as a catalyst for physical movement” (Greenbaum, 2006, *p*. 1). Within crisis settings, AACR has also been described as supporting first responders by providing opportunities for brief moments of decompression and self-care (Eaton-Stull & Flynn, 2015; Lackey et al., 2021). For trauma survivors, interactions with therapy dogs have been described as offering a non-judgmental source of comfort (Eaton-Stull, 2014; Fatjó & Bowen, 2023) and, in some cases, serving as a “vehicle for projection” through which individuals can express complex emotional experiences (Graham, 2009).

Although literature specifically examining AACR remains limited, a broader body of research on animal-assisted interventions (AAIs) provides contextual evidence regarding the potential role of human-animal interaction in trauma recovery. Studies involving therapy dogs and other AAIs have suggested improvements in PTSD symptoms and psychological well-being (Beetz, 2017; Germain et al., 2018; Mims & Waddell, 2016), as well as physiological indicators associated with reduced stress, although these findings cannot be directly generalized to AACR (Chandler, 2008; Lass-Hennemann et al., 2018; Robino et al., 2022). AACR differs from many other AAIs in its timing, setting, and conditions of delivery, as it is implemented in the immediate aftermath of crises and disasters and may occur in unpredictable and uncontrolled environments. Consequently, evidence from the wider AAI literature should be interpreted cautiously when considering AACR in acute trauma settings.

### 1.3. Gaps in the existing literature

Literature concerning the use of therapy animals in trauma settings remains largely descriptive or case-based (Altschuler, 2018), highlighting the need for additional empirical research on AACR (Lackey et al., 2021). Qualitative investigation is similarly limited, in part because conducting research with trauma survivors presents ethical and practical challenges (Jaffe, 2022). Consequently, several important aspects of AACR remain underexplored. For example, although relational dynamics appear to be central to the intervention, they have received limited empirical attention (Eaton-Stull et al., 2024). Similarly, the role of non-verbal communication within AACR remains poorly understood (Jaffe, 2022). Although AACR has also been described as benefiting first responders and bystanders, these observations have received little systematic empirical examination (Graham, 2009). In addition, cultural considerations influencing AACR implementation remain underexplored.

Consequently, little is known about how crisis responders experience and implement AACR during acute trauma response. This study addresses that gap by exploring responders' perspectives and experiences of implementing AACR during acute trauma response.

Although the use of AACR in acute trauma settings is increasing, the empirical evidence base remains limited, and qualitative research is particularly scarce. This study therefore explored crisis responders' perspectives and experiences of implementing AACR during acute trauma response. Specifically, it examined how responders perceived AACR to influence engagement, psychosocial support, and crisis response processes at trauma scenes in comparison with standard crisis intervention. Understanding responders' perspectives can contribute to the evidence base informing the implementation, refinement, and evaluation of trauma-informed psychosocial interventions in acute trauma settings. Throughout this paper, “standard crisis intervention” refers to psychosocial crisis response delivered without the inclusion of an AACR team.

## 2. Methods

### 2.1. Sample population

The sample consisted of 21 crisis responders from a national crisis response organization. Participants included mental health professionals, supervisors, and Psycho-Trauma First Responders**—**crisis responders who completed specialized training provided by the organization to provide immediate psychosocial support following traumatic events. Supervisors were responsible for supervising the organization’s Psycho-Trauma First Responder unit. Participants either implemented AACR during crisis response or worked alongside AACR teams during trauma response, and were therefore well positioned to describe their experiences and perspectives regarding its implementation in acute trauma settings. Participants had 3–25 years of crisis-response experience and 3–21 years of experience working with AACR. All interviews were conducted by the researcher, who was a PhD student at the time of the study and had professional experience as a mental health Animal-Assisted Crisis Responder.

Participant characteristics are presented in Table I. Of the 21 participants, five directly implemented AACR during crisis response, while 16 had experience working alongside AACR teams. The sample included participants with and without mental health professional backgrounds and represented a range of crisis-response and AACR experience.

**Table I.** Participant characteristics relevant to crisis response and AACR experience.

Characteristic	*n* (%) or M (SD)	Range
**Sex**		
Women	11 (52.4%)	–
Men	10 (47.6%)	–
**Professional background of the Crisis Responder**		
Mental health professional background	16 (76.2%)	–
No mental health professional background	5 (23.8%)	–
**Relationship to AACR**		
Directly implemented AACR	5 (23.8%)	–
Worked alongside AACR teams	16 (76.2%)	–
Crisis-response experience, years	8.4 (6.7)	3–25
Experience working with AACR, years	5.7 (5.0)	3–21

Note: *AACR = Animal-Assisted Crisis Response; M = mean; SD = standard deviation.*

Prior to data collection, the study received an exempt determination from an Institutional Review Board (IRB) under the U.S. Department of Health and Human Services Federal Policy for the Protection of Human Subjects (Common Rule), 45 CFR §46.104(d)(2). The exemption was granted for research involving interview procedures with adequate provisions to protect participant privacy and maintain data confidentiality.

Potential participants with relevant first-hand experience of crisis response and AACR were identified by the researcher and contacted by email with information about the study and an invitation to participate. Twenty-four individuals were approached, of whom 21 voluntarily agreed to participate. Participants completed semi-structured interviews by telephone, videoconference, or in person, according to their preference. Before each interview, verbal informed consent was obtained and documented in accordance with the approved study protocol. At the beginning of each interview, the interviewer read the informed consent information aloud, and participants provided verbal consent before the interview commenced. All participants granted permission for the use of recording devices during the interviews. Verbal consent was documented through the recording of each interview. Participation was voluntary, participants were informed of their right to withdraw from the study at any time without penalty, and all reasonable measures were taken to protect participant privacy and maintain the confidentiality of identifiable information throughout the research process. Participants received a restaurant voucher as a token of appreciation for their participation.

### 2.2. Data collection

Data collection consisted of a brief demographic questionnaire that collected information on professional background, role in crisis response and AACR, gender, religious practice, background regarding acceptance of dogs, and years of crisis-response and AACR experience, followed by semi-structured interviews. An interview guide comprising 17 open-ended questions explored participants' experiences of AACR, perceptions of its implementation in crisis settings, the populations for whom it was considered appropriate, and its perceived role during trauma response. Questions addressed participants’ initial experiences with AACR, perceived differences between crisis scenes with and without AACR, interactions with survivors and professionals, perceived benefits and challenges, populations considered more or less likely to benefit, and examples of situations in which AACR was perceived as helpful or potentially problematic. Interviews were guided by the interview schedule while allowing participants to elaborate on issues they considered important to their experiences.

### 2.3. Data analysis

All interviews were transcribed by the researcher with assistance from two students. The researcher uploaded the transcripts to ATLAS.ti Scientific Software Development GmbH (2022). An anonymized coding system was used to ensure confidentiality. Data were analyzed by the researcher using Braun and Clarke (2006, 2021) reflexive thematic analysis following an inductive approach. Coding began with close reading of each transcript to identify patterns of meaning. Initial codes were generated inductively and refined through continued engagement with the data. Codes were then examined across interviews and developed into broader themes, which were reviewed and refined in relation to the dataset. Reflexive memos documented developing interpretations and analytic decisions throughout this process. ATLAS.ti Scientific Software Development GmbH (2022) was used to organize transcripts, manage coding, support theme development, and retrieve illustrative quotations.

## 3. Results

Four themes were generated through reflexive thematic analysis that reflected participants' perceptions and experiences of implementing AACR during acute trauma response. An overview of the four themes identified through the analysis is presented in Table II.

**Table II.** Overview of themes and subthemes identified in the analysis.

Theme	Subtheme	Brief description
1. Perceived Relational and Non-Verbal Engagement Through AACR	–	AACR was perceived as facilitating rapport, reducing barriers to engagement, and creating opportunities for survivor-initiated and non-verbal connection.
2. Perceived Emotional Regulation and Crisis Stabilization	–	AACR was perceived as supporting emotional regulation, grounding, and stabilization through physical presence, sensory engagement, and redirection of attention.
3. Perceived Influence on Crisis Response Dynamics	–	AACR was described as influencing the emotional and interpersonal dynamics of crisis scenes and facilitating dialog, emotional expression, psychoeducation, and a sense of control.
4. Contextual Considerations in AACR Implementation	4.1 Settings and Populations for AACR	Participants described settings and populations for which AACR was perceived as particularly relevant, including diverse trauma contexts and groups with differing communication and emotional needs.
	4.2 Operational, Cultural, and Welfare Considerations	Participants identified contextual factors affecting AACR implementation, including scene suitability, logistical and institutional constraints, cultural considerations, and the welfare of AACR dogs and responders.

### 3.1. Perceived relational and non-verbal engagement through AACR

AACR teams were described as transforming the emotional atmosphere at trauma scenes, fostering greater openness and facilitating survivor-initiated engagement. Compared to standard crisis intervention approaches, both survivors and first responders were more willing to approach AACR teams. This was evident among firefighters who initially appeared emotionally closed but became more open to conversation after interacting with the AACR dog: *“The next thing we knew, they were talking to the therapist about how tough it was for them.”* (Participant 3, mental health crisis responder). A similar shift was noted among responders who were initially hesitant about introducing AACR into trauma scenes or working alongside an AACR team but reported changing their perceptions after direct experience with the intervention: *“I was quite wary of the idea, to begin with… but I very quickly warmed up to the therapy dog.”* (Participant 7, mental health crisis responder supervisor). Over time, AACR shifted perceptions of the intervention and increased recognition of its value among responders.

Participants described AACR as creating natural opportunities for emotional connection and intervention: *“the animal is the 'key' that joins the survivor and the crisis responder.”* (Participant 7, mental health crisis responder supervisor). AACR was described as facilitating rapport-building and creating informal, emotionally accessible engagement pathways at trauma scenes. For example, one interviewee explained that in some trauma scenes *“nobody wants to speak to therapists.”* (Participant 3, mental health crisis responder). Through AACR intervention, *“you don't need to be flagged and tagged as a patient”* to take part in the interaction (Participant 6, crisis responder). Survivors were described as initiating contact, inviting AACR handlers to sit with them, and seeking support from responders. AACR was also perceived as reducing survivors’ defensiveness and increasing openness to interaction. For the survivors, *“there was no reason to put up a wall.”* (Participant 3, mental health crisis responder).

### 3.2. Perceived emotional regulation and crisis stabilization

AACR appeared to help reduce stress and anxiety through touch, comfort, physical presence, and emotional support during crises: *“Human touch is so important but needs permission; animal touch doesn't.”* (Participant 7, mental health crisis responder supervisor). AACR’s presence was perceived as calming and supportive without requiring verbal interaction: *“People could just be with the animal.”* (Participant 1, crisis responder). Participants perceived AACR as fostering feelings of safety during crises, supporting stabilization and recovery. AACR was also observed to alleviate acute distress and support emotional stabilization during crises: “[The AACR dog] helped us distract people and also helped us focus people.” (Participant 5, mental health crisis responder supervisor). By momentarily redirecting attention, AACR was perceived as supporting stabilization and engagement at trauma scenes. Responders also described AACR as facilitating grounding through sensory and physical connection, helping survivors return their attention to the present moment. One responder described AACR as *“bringing the person back to the present.”* (Participant 4, mental health crisis responder supervisor).

### 3.3. Perceived influence on crisis response dynamics

AACR was described as altering the emotional and social atmosphere, and introducing moments of lightness, distraction, and interpersonal engagement. Described as *“bringing something different and special”* (Participant 4, mental health crisis responder supervisor) that *“changed the dynamics”* (Participant 5, mental health crisis responder supervisor) of the scene, AACR was described as providing survivors with an opportunity to *“disconnect for a few seconds from the chaos.”* (Participant 5, mental health crisis responder supervisor). This temporary shift appeared to create immediate opportunities for emotional relief, facilitating emotional and interpersonal changes among survivors: *“In a matter of minutes, she (a bystander) was smiling and laughing”* (Participant 2, mental health crisis responder). Furthermore, mental health responders perceived AACR as a more relational, calming, and *“gentle and easier”* (Participant 2, mental health crisis responder) intervention compared to standard crisis response, describing greater opportunities for dialog, easier verbal expression of traumatic experiences, and interactions that developed more naturally. They also perceived the presence of AACR as contributing to a calmer and less emotionally heavy atmosphere at the trauma scene.

A central narrative among participants was the AACR dog’s perceived ability to intuitively identify survivors in distress, with one responder highlighting *“the dog's ability to sense the needs of trauma survivors.”* (Participant 14, mental health crisis responder). This capacity was often manifested when the AACR dog intuitively initiated contact with survivors: *“[The AACR dog] shoves its head against them so the survivors pet the dog.”* (Participant 3, mental health crisis responder). Responders described this as allowing them to assess and navigate complex scenes more efficiently while gaining access to emotionally vulnerable survivors.

Once a connection was established, participants described AACR as acting as a *“catalyst for change,”* facilitating emotional release, a greater sense of control, and verbal emotional expression (Participant 1, crisis responder). Interviewees emphasized that survivors often used AACR interactions to share how they felt, vent emotionally, and unburden themselves during crises. Furthermore, AACR was described as allowing survivors to project emotions and experiences onto the animal, facilitating indirect emotional expression during crisis intervention: *“All these questions he [the survivor] would project via the animals.”* (Participant 19, mental health crisis responder). Concurrently, responders leveraged the animal’s behavior under stress to deliver immediate psychoeducation, helping survivors understand and normalize their own reactions. For example, responders described using an animal’s shaking when stressed to explain that people may also experience physical reactions to stress, helping survivors understand such responses as normal reactions to a traumatic event. Interviewees emphasized that AACR intervention supported normalization by *“bringing the person back to normal in a place where there was chaos.”* (Participant 4, mental health crisis responder supervisor). AACR was perceived as helping survivors regain a sense of control during crises, often through active interaction with the AACR dog, such as holding the leash.

### 3.4. Contextual considerations in AACR implementation

#### 3.4.1. Settings and populations for AACR

Participants identified AACR as particularly relevant across trauma settings involving disasters, fires, sudden deaths, mass-casualty events, and terror attacks, as well as in shelters and scenes involving bystanders, such as children and youth. One responder explained that AACR *“has been extremely useful in the different kinds of human-made mass casualties”* (Participant 9, crisis responder supervisor). It was identified as especially valuable in trauma settings requiring emotional stabilization, grounding, and rapid engagement.

Participants perceived AACR as particularly beneficial for children, non-verbal survivors, individuals who were guarded or resistant to interpersonal connection, youth at risk, adults during war, and elderly survivors who struggle with verbal emotional processing. One participant described considering AACR particularly in situations “involving kids” and “non-verbal patients,” including individuals with special needs, older adults, or those experiencing language barriers (Participant 6, crisis responder). Another participant described AACR as particularly beneficial for individuals who “feel guarded to verbal expression and emotional vulnerability” or are “resistant to connecting with other humans in an emotionally vulnerable way” (Participant 4, mental health crisis responder supervisor). Interviewees emphasized AACR's ability to facilitate non-verbal communication, emotional safety, and emotional expression across different populations. AACR dogs were described as *“a walking piece of play therapy”* (Participant 3, mental health crisis responder).

Narratives indicated that AACR was perceived as beneficial not only for trauma survivors but also for bystanders, first responders, mental health responders, and AACR responders themselves: *“They'll just pet, hug, spend their few minutes with the animals”* (Participant 9, crisis responder supervisor). Participants emphasized AACR's perceived relevance across multiple groups present at trauma scenes.

#### 3.4.2. Operational, cultural, and welfare considerations

Despite its perceived relevance across diverse settings and populations, participants emphasized that AACR was not appropriate for all crisis environments and required careful evaluation of risks and benefits: *“One can't bring an animal into every scene.”* (Participant 13, mental health crisis responder). Consequently, scene assessment is essential before deployment. Participants described AACR as potentially appropriate across diverse settings, including fires, natural disasters, mass-casualty incidents, and more individualized trauma scenes such as sudden deaths and car accidents. In contrast, highly hectic or unsafe environments were considered less appropriate when the presence of the dog could add complexity, increase stress, or place the animal at risk. For example, AACR responders described avoiding deployment to some rocket-related crisis scenes because of concerns for the dog’s safety. These considerations were described as part of a broader decision-making process regarding whether and how AACR teams should be deployed. Deploying AACR teams involves greater organizational, logistical, and administrative complexity than standard crisis response, often requiring additional approval and decision-making processes. Institutional restrictions and access barriers were also described as challenges to AACR intervention at trauma scenes.

Even when logistically feasible, participants additionally emphasized the importance of cultural and religious sensitivity when implementing AACR interventions. Survivors’ fear of dogs, limited prior exposure to animals, and specific traditional beliefs regarding dogs as ‘unholy animals’ in some Middle Eastern communities can act as barriers to AACR intervention. These factors can restrict the implementation of AACR in certain situations.

Across interviews, the importance of selecting, training, and protecting AACR dogs during crisis intervention was emphasized: *“Safety is the first rule in crisis response.”* (Participant 6, crisis responder). Responders described animal welfare, stress monitoring, and scene appropriateness as essential considerations in AACR implementation. One AACR responder illustrated how the dog’s needs could require the handler to pause during crisis response: *“She may have the strength while the dog may not. She might have to stop to feed the dog or let the dog rest even though she is energetic and able to go on.”* (Participant 3, animal-assisted crisis responder). AACR handlers also described the intervention as requiring simultaneous attention to the trauma scene, the survivor, and the animal: *“The handler must be a multitasker”* (Participant 12, mental health crisis responder).

Furthermore, the intervention was described as emotionally demanding due to repeated exposure to trauma scenes and concern for the AACR dog’s well-being. Interviewees emphasized the importance of monitoring handlers’ emotional exposure during crisis response, while also describing the AACR dog's presence as emotionally supportive during traumatic interventions. This dual role was reflected in participants’ descriptions of the dog as both a responsibility during crisis response and a potential source of support for the responder. As one participant explained, *“Holding and petting the dog really heals me”* (Participant 13, animal-assisted crisis responder).

## 4. Discussion

The findings suggest that responders perceived AACR as influencing acute trauma scene dynamics by facilitating engagement, emotional stabilization, and grounding. These findings extend the emerging literature on trauma-informed psychosocial interventions during acute crisis response.

### 4.1. Perceived relational and non-verbal engagement through AACR

A primary contribution of this study is the refinement of how AACR was perceived to facilitate rapport. While prior literature notes that AACR dogs may often act as a bridge of connection between trauma survivors and responders (Chandler, 2008; Greenbaum, 2006), the current findings extend this concept by emphasizing AACR’s role in creating a non-threatening and survivor-initiated pathway into crisis intervention. Trauma survivors may reach out to the AACR team instead of the mental health responder approaching them (Chandler, 2008; Graham, 2009). This is particularly notable among individuals who struggle with direct human contact or verbal engagement, including children and survivors with avoidance-related difficulties. According to Graham (2009, p.76), “the dog serves as an entrée for the establishment of a relationship and a venue for the affected individuals to talk,” suggesting the AACR team may function as an effective icebreaker for therapeutic engagement. The current findings further support this idea by suggesting that AACR may create a unique first point of contact that encourages openness to intervention.

This mechanism may be especially relevant for individuals who cannot tolerate direct human contact or struggle with verbal processing during acute distress but may be willing to interact with an animal (Greenbaum, 2006). AACR's relational and non-verbal qualities can also be understood through biophilia theory, which emphasizes humans' tendency to connect with other living organisms (Wilson, 1984). Within this framework, the nonjudgmental and non-threatening presence of AACR dogs may help lower survivors' barriers to engagement following a traumatic event and serve as relational bridges during crisis intervention.

### 4.2. Perceived coping, normalization, and recovery processes

The present findings suggest that responders perceived AACR as supporting immediate stabilization by redirecting survivors’ attention, facilitating grounding, and helping them return their attention to the present moment. These findings are consistent with previous literature describing the AACR dog's potential contribution to survivors’ sense of safety (Eaton-Stull, 2014) and the use of the dog's presence to redirect attention away from the traumatic environment (Graham, 2009; Greenbaum, 2006).

Beyond immediate stabilization, the present findings suggest that AACR may support coping by providing opportunities for emotional expression, projection, psychoeducation, and normalization during acute crisis response. A central process identified in this study is the AACR dog’s function as a safe, symbolic medium onto which survivors can project their overwhelming emotions, fears, and acute stress experiences (Bua, 2013). This capacity for projection expands upon existing AACR literature on projection and psychoeducation (Graham, 2009; Greenbaum, 2006; Stewart et al., 2016) by demonstrating how the animal allows for indirect emotional experience and facilitates processing. The findings suggest that interacting with the AACR dog may provide survivors with an opportunity to express complex trauma-related emotions without requiring direct self-disclosure. Consequently, the findings suggest that AACR may create emotionally accessible pathways for trauma survivors who may struggle with direct verbal processing during crisis, showing more willingness to vent, unburden themselves, and communicate emotionally while interacting with the animal.

Moreover, the findings suggest that AACR may support survivors through normalization and a greater sense of control during crisis response. Participants described active interaction with the AACR dog, such as holding the leash, as providing survivors with an opportunity to participate rather than remain passive during the crisis response. This perceived restoration of control may represent one way in which AACR supports coping during acute distress. According to Antonovsky, (1987), individuals respond differently to traumatic events based on their baseline resilience. While some cope naturally, others need interventions that normalize their reactions to traumatic situations (Greene & Greene, 2009). The current findings highlight that AACR may facilitate this normalization process, consistent with previous literature (Greenbaum, 2006; Graham, 2009).

### 4.3. Perceived contribution to standard crisis response

The present findings suggest that AACR may complement standard crisis response by facilitating engagement and temporarily redirecting survivors’ attention away from the traumatic environment. This is consistent with previous literature suggesting that therapy animals may serve as catalysts for engagement and early recovery following traumatic events (Greenbaum, 2006; Wells, 2009).

Furthermore, AACR teams were described as facilitating access to and engagement with emotionally vulnerable survivors during acute stress (Bua, 2013; Greenbaum, 2006; Stewart et al., 2016). Overall, AACR was perceived as complementing standard crisis intervention. AACR teams were generally described as being well received by both first responders and survivors at trauma scenes.

Chandler (2008) compared AACR to standard crisis response in the context of the Hurricane Katrina crisis, highlighting the role of integrating therapy dogs in reducing anxiety and providing essential comfort to both survivors and first responders. The current findings extend this literature by suggesting that AACR may offer unique relational and emotional qualities during crisis intervention at trauma scenes. Touch and proximity to the animal were perceived as helping to lower stress during AACR intervention (Eaton-Stull & Flynn, 2015). Previous literature suggests that touch may support parasympathetic nervous system regulation (Shubert, 2012; Lackey et al., 2021), while also providing comfort, nurturing interaction (Graham, 2009), and grounding (Greenbaum, 2006; Stewart et al., 2016). The AACR dog was also perceived as a compassionate and grounding presence during crisis intervention (Eaton-Stull & Flynn, 2015; Greenbaum, 2006). Taken together, these findings suggest a potential role for AACR in supporting grounding during acute crisis response.

### 4.4. Operational and contextual complexities of AACR

The present findings emphasize that the appropriateness of AACR depends on careful assessment of the specific crisis environment, including potential risks to survivors, responders, and the AACR dog. Participants described scene suitability, safety, and operational demands as important considerations when determining whether AACR should be deployed. These findings are consistent with Greenbaum (2006) emphasis on AACR team preparation and careful scene assessment.

While the existing literature discusses the need for appropriate training of therapy animals (Bua, 2013; Lackey et al., 2021), this study underscores that AACR intervention occurs within a highly demanding operational environment for both handlers and their dogs. AACR responders discussed how they personally benefited from having their dog with them at trauma scenes. However, some mentioned the potential stress associated with caring for the AACR dog during a trauma scene. Graham (2009) emphasizes the importance of handlers remaining attentive to the dog's stress level and taking breaks when necessary. Thus, even when a dog is appropriately selected and trained for AACR, ongoing attention to its welfare, safety, and stress levels remains essential.

The current study suggests that prior exposure and subjective affinity toward dogs may be important factors in determining the suitability of AACR for individual survivors. Responders also described AACR dogs as creating opportunities to introduce dogs to individuals who were not accustomed to them. While Greenbaum (2006) noted the limiting impact of phobias, superstitions and socio-cultural beliefs against canines, previous studies have not fully addressed how survivors’ baseline familiarity and affinity toward dogs influence their openness to AACR intervention. The current findings suggest that considering these pre-existing individual and cultural factors may help determine the appropriateness of AACR while reducing the potential for secondary distress during crisis intervention. Our findings further highlight the relationship between the AACR teams and the communities they serve, including familiarity with local culture and context, which may support engagement within specific community systems. This emphasis on cultural context is consistent with previous literature highlighting the importance of considering the cultural backgrounds of individuals present at trauma scenes (Greenbaum, 2006; Stewart et al., 2016) and understanding survivors within their broader community context (Kilmer & Gil-Rivas, 2010; Norris et al., 2008). Overall, AACR was described as a valuable addition to crisis intervention, while emphasizing the importance of culturally sensitive implementation.

### 4.5. AACR within varied populations and settings

The present findings suggest that responders perceived AACR as relevant across a range of trauma settings and populations, including children, non-verbal survivors, individuals who were guarded or resistant to interpersonal connection, older adults, bystanders, and first responders. Participants particularly emphasized AACR's perceived value for individuals who may have difficulty with verbal emotional processing, suggesting that its non-verbal and relational qualities may broaden opportunities for engagement across different populations. These findings are consistent with previous literature describing the potential value of AACR for children (Chandler, 2008), first responders (Shubert, 2012), mental health professionals (Stewart et al., 2016), and survivors' families (Greenbaum, 2006; Shubert, 2012).

Differences also emerged between the trauma settings described in the U.S. literature and those reflected in the present findings. While international literature typically frames AACR within the context of mass-casualty events or large-scale community disasters (Eaton-Stull et al., 2010; Lackey et al., 2021), participants in the present study described its deployment across a broader range of settings, including small-scale, highly intimate crises such as apartment fires, the sudden death of a family member, missing-person incidents, and motor vehicle accidents. These smaller and more intimate scenes may nevertheless present substantial traumatic risk for the individuals and communities affected. These findings contribute to the emerging qualitative evidence base on trauma-informed psychosocial interventions in acute trauma settings.

### 4.6. Implications

This study contributes qualitative insights that may inform the continued development and evaluation of AACR within crisis response practice. The findings suggest that responders perceived AACR as complementing standard crisis intervention through relational and non-verbal mechanisms that supported emotional stabilization, engagement, grounding, and communication during traumatic events. These findings may be relevant for practitioners already working within animal-assisted interventions who are considering AACR training to expand their therapeutic repertoire. Moreover, the findings highlight the importance of developing culturally and contextually sensitive AACR protocols for crisis response. The findings further highlight the value of immediate intervention at trauma scenes, as well as the integration of mental health professionals within AACR teams. Furthermore, training in immediate trauma intervention requires the ability to address relevant trauma-informed principles, such as cultural competence, sensitivity, and improvisation, while considering interdisciplinary crisis response and the welfare of the AACR dogs.

### 4.7. Limitations of the study

Several methodological limitations should be discussed. First, data were collected from crisis responders who directly implemented AACR as well as responders who had experience working alongside AACR teams during trauma response, rather than from trauma survivors directly receiving the intervention. Although this provided professional perspectives regarding AACR implementation and crisis response dynamics, survivor experiences were not directly explored. Another limitation concerns the sample size and recruitment strategy, as all participants were members of the same organization. The relatively small sample may have limited the range of responder perspectives and experiences represented in the study. Recruiting participants from a single organization may have influenced perspectives on AACR and introduced potential organizational bias. Finally, the researcher’s role as an Animal-Assisted Crisis Responder may have influenced the interpretation of the data. This professional experience provided familiarity with AACR and the crisis-response context, while also creating the potential for prior experiences and assumptions about AACR to shape attention to and interpretation of participants’ accounts. To enhance reflexivity, analytic memos were maintained throughout coding and themes were refined through repeated review of the interview data. Accordingly, the findings may not be transferable to all crisis response organizations or cultural contexts.

### 4.8. Future research

Future research should further investigate the experiences of trauma survivors and bystanders directly receiving AACR intervention in crisis settings. Because the present study examined AACR primarily from the perspectives of crisis responders, research involving survivors and bystanders could clarify whether the relational, emotional, and stabilizing processes perceived by responders are similarly experienced by those receiving the intervention.

Additional research is needed regarding the experiences of AACR responders, the emotional and operational impact of crisis intervention on therapy animals, and the interaction between handler and animal welfare during crisis deployment. Such research could help clarify the demands placed on AACR teams and inform decisions regarding training, deployment, breaks, and scene suitability.

Future research should also examine the contextual factors that may influence whether AACR is appropriate for a particular crisis scene, including cultural familiarity with dogs, prior exposure to animals, scene complexity, and operational demands. Examining these factors across different crisis-response organizations and cultural contexts could help identify circumstances in which AACR is perceived as appropriate as well as those in which its use may be limited.

## 5. Conclusion

This research advances understanding of AACR from the perspective of crisis responders involved in acute trauma response. The findings suggest that responders perceive AACR as a valuable component of trauma-informed psychosocial crisis response. Further research is needed regarding survivor experiences, responder well-being, animal welfare, and culturally responsive AACR implementation. Rather than demonstrating intervention effectiveness, this study provides qualitative insight into how crisis responders perceived the implementation and role of AACR during acute trauma response while identifying priorities for future implementation, evaluation, and research.

## Supplementary Material

Supplementary Material Informed Consent.docx

## Data Availability

The data supporting the findings of this study are not publicly available because they contain information that could compromise participant confidentiality. De-identified data may be available from the corresponding author upon reasonable request and subject to institutional ethics approval.
