# Supplementary material for: Crisis responders' perspectives on animal-assisted crisis response in acute trauma settings: a qualitative study
Source: Int J Qual Stud Health Well-being. 2026 Sep 10;21(1):2732305. doi: 10.1080/17482631.2026.2732305 (PMC13564164; doi:10.1080/17482631.2026.2732305)
Supplement: Supplementary file 1 [file ZQHW_A_2732305_SM2365.docx]

**INFORMED CONSENT FORM**

I voluntarily agree to participate in the research study titled:

*“Animal-Assisted Crisis Response in Israel”*

          The proposed research project will be undertaken by Batya G Jaffe a doctoral candidate at Wurzweiler School of Social Work, Yeshiva University, New York, New York under the direction of Dr. Susan E. Mason.

I entirely recognize that my intention to participate in this research project is voluntary. I can refuse to participate or stop taking part at any time without giving any reason. Also, I understand that I can demand for the return of all information about me and/or have them deleted at any time and stage of the research process.

The reason for the proposed study is to understand and document the value of the contribution of Animal-Assisted Crisis Response (AACR) to the people affected in the aftermath of trauma in Israel. The specific aims of this study are to explore how AACR affects the survivors, the bystanders, and the responders on the scene of a traumatic event through interviews conducted with the mental health professionals on the scene. A secondary aim is to explore the individual experience of responders with AACR.

I understand that if I volunteer to partake in the proposed research project, I may be required to:

• To participate in a virtual interview conducted using the software, Zoom or a telephone interview.

• I understand that the interview data will be kept as confidential and participants will not be identified in any publication or report resulting from this study.

No risk is expected from your participation.

To further enhance confidentiality, all electronic data relating to participants for the proposed research project will be safe guided under lock and key of my filing cabinet; those stored in my working computer/portable hard drives will be password protected.

If you volunteer to participate you may decide to discontinue at any time during the study by simply exiting the interview. By doing so all information you provided will be digitally deleted. You are also free to skip question/s.

Your participation is entirely voluntary, and the researcher will answer any further questions regarding the proposed study throughout the study period, and can be contacted at (972) 54-819-2707 or by email at bjaffe2@mail.yu.edu.

You may also contact the study supervisor, Dr. Susan E. Mason at 1-646-592-6806 or Email: masonse@yu.edu

By participating in this interview, I am agreeing to voluntarily taking part in this study project.

Also, you may print and retain a copy of the informed consent form for your records.

PLEASE SIGN:_________________________________
